# Reversible Switching and Recycling of Thermoresponsive 1,2,4-Triazolium-Based Poly(ionic liquid) Catalysts for Porous Organic Cage Synthesis in Organic Media

**DOI:** 10.1021/acsmacrolett.5c00072

**Published:** 2025-03-24

**Authors:** Jiefeng Zhu, Feng Chen, Jie Zhang, Ruijie Hou, Jian-ke Sun, Xianjing Zhou, Jiayin Yuan, Xinping Wang

**Affiliations:** † School of Chemistry and Chemical Engineering, Key Laboratory of Surface and Interface Science of Polymer Materials of Zhejiang Province, Zhejiang Sci-Tech University, Hangzhou 310018, China; ‡ State Key Laboratory for Modification of Chemical Fibers and Polymer Materials, College of Materials Science and Engineering, Donghua University, Shanghai 201620, China; § School of Chemistry and Chemical Engineering, Beijing Institute of Technology, Beijing, 102488, China; ∥ Department of Chemistry, Stockholm University, Stockholm 10691, Sweden

## Abstract

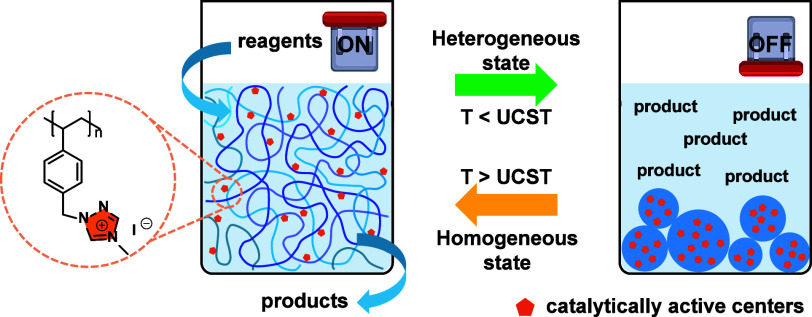

Homogeneous catalysts of high activity and selectivity often face challenges in the separation from feedstocks and products after reactions. In contrast, heterogeneous catalysts are easier to separate, usually at the cost of compromised catalytic performance. By designing catalysts capable of switching between homogeneous and heterogeneous states for catalysis and separation, the merits of both could be synergistically combined. In this study, a thermoresponsive 1,2,4-triazolium-based poly(ionic liquid) (PIL) was applied as a temperature-switchable organocatalyst for the controlled synthesis of porous organic cages in methanol. Variation of the reaction temperature induced a phase transition of the PIL, causing the polymer chains to dissolve or collapse in methanol, thereby exposing or shielding the catalytically active sites to proceed or retard the reaction, respectively. To note, at a sufficiently low temperature, the PIL as a catalyst precipitated out of its methanol solution and could be separated by centrifugation or filtration for reuse, similar to common heterogeneous catalysts. Such switchable and recyclable properties of polymeric catalysts will inspire the design of efficient and adaptable organic or hybrid nanoreactors in liquid media.

Catalysts play a pivotal role in modern chemical technologies, environmental remediation, biological systems and more, with their significance spanning numerous scientific, industrial, and biological contexts.^1−3^ Homogeneous catalysts, in their nature, are typically equipped with higher activity and/or selectivity than heterogeneous catalysts. However, a major drawback of homogeneous catalysts is the difficulty in their efficient separation from feedstocks and products in the reaction mixture, which limits their use in real life. In contrast, heterogeneous catalysts are usually more stable and can be readily isolated from the reaction mixture for reuse.^4,5^ Developing catalysts that can seamlessly switch between homogeneous and heterogeneous states without a change in their chemical nature offers a promise to combine the merits of both systems–achieving efficient catalysis in a homogeneous state while enabling straightforward separation and recovery of the catalysts in a heterogeneous state for reuse.

Stimuli-responsive polymers, also known as “smart” polymers, undergo a drastic and reversible variation in their physical or chemical properties, including molecular conformation, macroscopic coloration, and solubility, in response to external stimuli, such as temperature, pH, and light.^6−13^ These polymers have recently garnered interest due to their capability of regulating mass transfer in heterogeneous catalysts, mimicking the operation of enzymes in a fully reversible manner. Due to their switchable properties in response to external stimuli, these systems are capable of regulating the transport of reactants and products in solutions, thus modulating reaction rates and toggling catalytic processes ON/OFF.^14−16^ Among “smart” materials, thermoresponsive polymers stand out for their unique thermal reversibility in solution states, where the most studied examples are neutral or weakly charged polymers in aqueous solutions.^17−21^ For instance, Albanese et al.^22^ grafted poly(*N*-isopropylacrylamide) onto a Pd/SiO_2_ surface and utilized its thermoresponsiveness to tune the extent of solvation of key surface reaction intermediates in the transition state during the hydrogenation of nitrobenzene to aniline. Liu and co-workers^23−25^ developed a series of dual-activity switchable catalysts using water-soluble thermoresponsive polymers to precisely manipulate cascade reactions. Chen et al.^26^ developed a continuous catalyst-recycling method using flow chemistry with a polymer-induced thermoresponsive catalyst for general, efficient, and a low Pd-loading Suzuki–Miyaura coupling reaction. Yin et al.^27^ developed a thermoresponsive poly(*N*-isopropylacrylamide)-supported chiral Salen manganese III catalyst that accelerated the asymmetric epoxidation of unfunctionalized olefins by thermoresponsive self-assembly of the catalyst in water.

Despite a bunch of examples, most systems utilize the thermal sensitivity of polymers to modulate catalytic performance in aqueous environments as these polymers are inherently thermoresponsive in water. There are fewer examples of thermoresponsive polymers being employed to modulate the catalytic performance in organic solvents. It is noteworthy that many chemical syntheses due to solubility issues are catalyzed in organic media. Recently, we discovered that a methyl-substituent 1,2,4-triazolium-based poly(ionic liquid) (PIL) with I^–^ as the counterion (termed Ptriaz-C1-I) exhibited tunable upper-critical-solution-temperature (UCST)-type phase transition in methanol.^28^ Porous organic cages (POCs) are a class of microporous materials with intrinsic, accessible cavities that are widely used in catalysis, storage, recognition, and separation.^29−33^ Sun et al.^34^ reported that poly(1,2,4-triazolium)s could serve as universal additives to accelerate by at least 1 order of magnitude the catalytic growth of representative imide-linked crystalline POCs.

Inspired by these pioneering studies, we applied the thermoresponsive 1,2,4-triazolium-based PIL as a switchable polymeric catalyst for the synthesis of POCs in methanol. Their catalytic activity was demonstrated to be adaptive in a reversible manner by adjusting the temperature (Figure 1). Above the temperature at UCST (*T*_UCST_), the catalytically active PIL was dissolved molecularly in methanol and, thus, acted as homogeneous catalysts to achieve high reaction rates. At the end of the reaction, the catalysis was “turned OFF” simply by cooling the reaction mixture solution below *T*_UCST_ to aggregate and precipitate the PIL chains. The precipitate was readily separated from the reaction mixture by centrifugation or filtration and was redissolved in a fresh reaction system for reuse as catalysts, a process that could be repeated.

**Figure 1:**
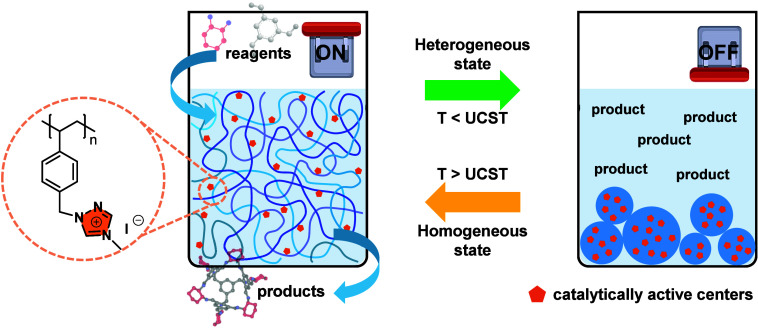
Schematic of a switchable thermoresponsive 1,2,4-triazolium-based PIL catalyst. The reaction is efficiently catalyzed at a temperature higher than *T*_UCST_ (ON) in a homogeneous state and deactivated upon cooling (OFF) below *T*_UCST_ into a heterogeneous state to separate the catalyst from the reaction mixture by centrifugation or filtration for reuse.

The rate-determining step in Schiff base condensation reactions is often the dehydration of the carbinolamine intermediate when aldehydes and amines react under typical conditions (e.g., in a weakly acidic environment). This step can be catalyzed by protons.^35^ Sun et al.^34^ demonstrated that 1,2,4-triazolium PILs sufficiently catalyzed the Schiff base condensation reaction. The strong solvation ability of the PIL chains enables them to outcompete solvent molecules in interacting with the solute, while the C5 proton of the 1,2,4-triazolium cation ring readily ionizes within the reaction system. These two phenomena act synergistically to promote the formation and subsequent dehydration of the carbinolamine intermediates,^34^ making 1,2,4-triazolium-based PILs highly efficient for catalyzing this reaction.

We first verified whether the 1,2,4-triazolium-based PIL with a methyl substituent and I^–^ as the counterion (termed Ptriaz-C1-I) could catalyze the growth of the imine-conjugated porous organic cage CC3R (Figure 2a). The synthesis of Ptriaz-C1-I and porous organic cage CC3R are detailed individually in our previous papers^28,36^ and briefly introduced in the Experimental Section in Supporting Information. The reaction product obtained at 60 °C in the presence of Ptriaz-C1-I at a concentration (*C*_PIL_) of 20 mg/mL, termed CC3R-P20-60, was analyzed as a representative model. Its powder X-ray diffraction (XRD) diagram is shown in Figure 2b, where both experimental and simulated data (in black in Figure 2b)^34,37^ are found consistent, suggesting the success in synthesizing the target porous organic cage CC3R. The chemical structure of CC3R was further confirmed based on the characteristic chemical shifts^38,39^ and their integral in its proton nuclear magnetic resonance (^1^H NMR) spectrum (Figure 2c). Its Fourier transform infrared (FT-IR) spectrum is shown in Figure 2d. The absorption bands at 1649, 1601, and 1342 cm^–1^ are attributed to the vibrations of −C=N–, −C=C–, and −C–N–, respectively.^37,40^ Next, the morphology of CC3R-P20-60 was characterized by optical microscopy, as shown in Figure 2e. Scanning electron microscopy (SEM) further revealed that the crystalline phase appears ortho-octahedral and consists of six vertices with eight ortho-triangles.^38,41^ The prism length is 5.6 ± 1.6 μm, as measured from Figure 2f,g.

**Figure 2:**
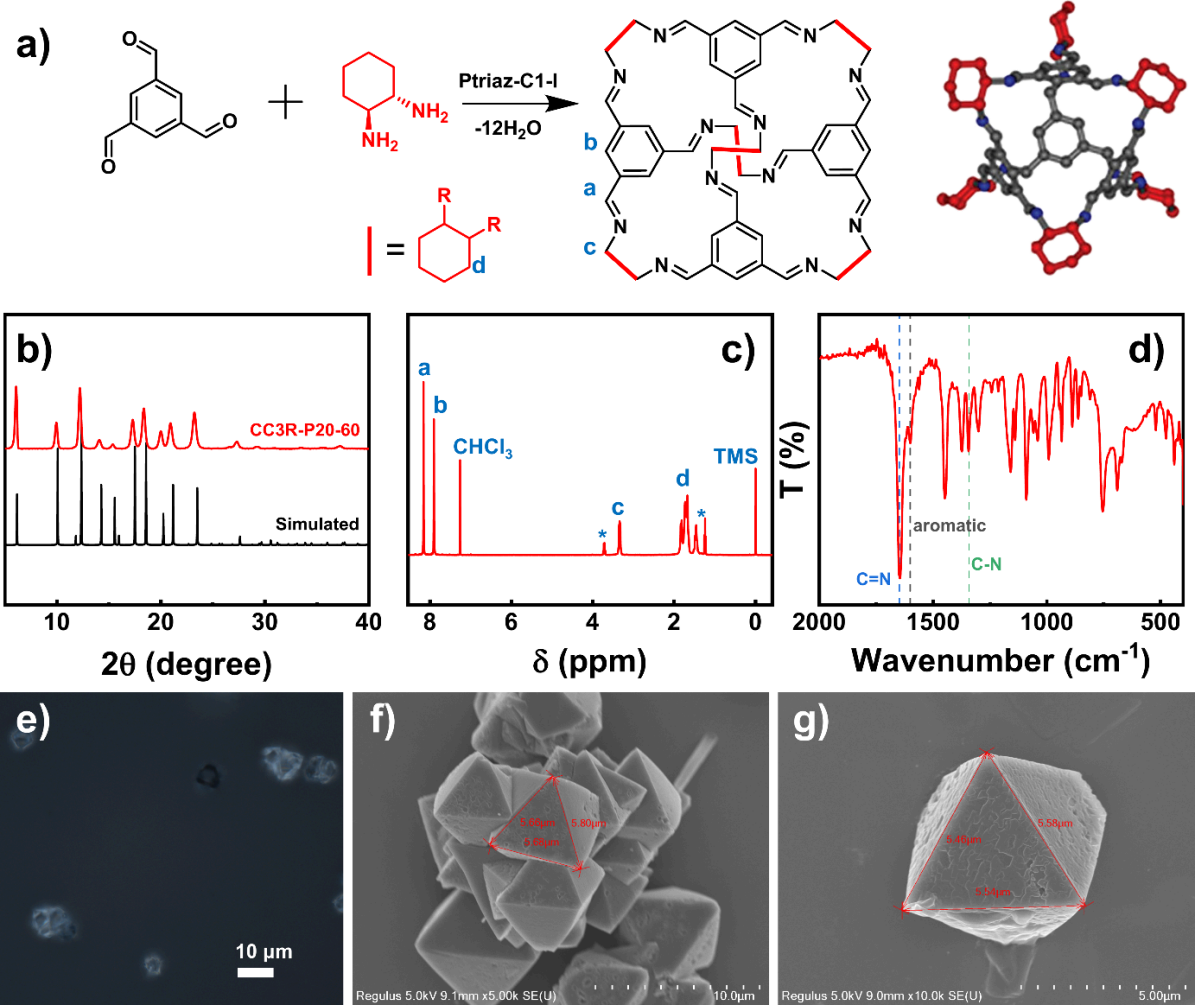
(a) Synthetic scheme of the porous organic cage CC3R, whose synthesis was catalyzed by Ptriaz-C1-I. (b) Powder XRD spectra of CC3R-P20-60 (red line) and simulated data^37^ (black line). (c) ^1^H NMR and (d) FT-IR spectra of CC3R-P20-60. (e) Representative image from reflection mode of optical microscope. (f and g) SEM photographs of CC3R-P20-60 in an overview and close view, respectively.

Since porous organic cages are a well-known class of adsorbent materials, nitrogen gas sorption was employed to characterize the porous structure of CC3R-P20-60. As shown in Figure S1, CC3R-P20-60 presents a classic type I isotherm with a specific surface area (*S*_BET_) of 501 m^2^g^–1^, as calculated by the Brunauer–Emmett–Teller (BET) equation. This value is comparable to common porous organic cages reported in the literature.^37,39,42^

Very recently it was discovered by us that 1,2,4-triazolium-based PILs, e.g., Ptriaz-C1-I in methanol underwent a UCST-type phase transition and showed low-temperature precipitation and high-temperature dissolution.^28^ Its phase diagram is shown in Figure 3a. Meanwhile, Ptriaz-C1-I has been identified as a good catalyst for the preparation of CC3R. Combining these two scenarios together, we hypothesized that thermoresponsive Ptriaz-C1-I could serve as “smart” catalyst to facilitate the synthesis and isolation of CC3R.

**Figure 3:**
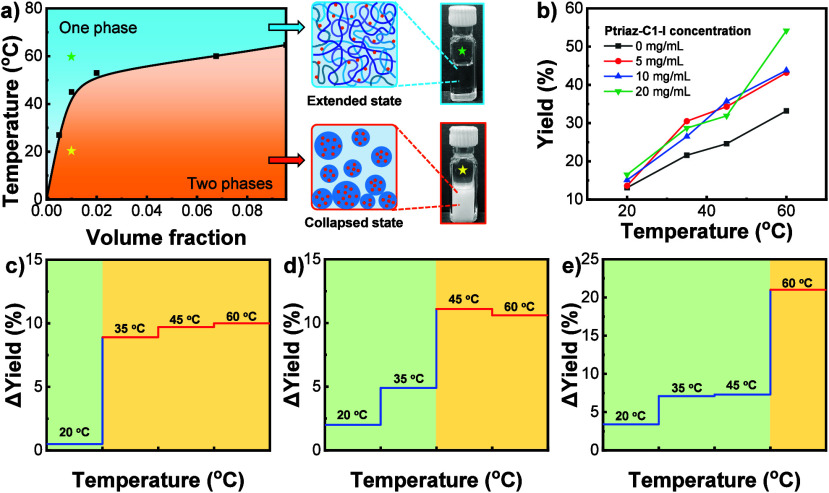
(a) Phase diagram of the Ptriaz-C1-I/methanol system and state of Ptriaz-C1-I chains. The photographs show the appearance of the solution at the green and yellow star markers in the phase diagram, respectively. *C*_PIL_ ∼ 10 mg/mL, 20 °C (bottom, <*T*_c_, yellow star) and 60 °C (top, >*T*_c_, green star). (b) Synthetic yield of CC3R obtained at different *C*_PIL_s for 1 h at varied temperatures from 20 to 60 °C. (c–e) Difference in yields of CC3R in the reaction with and without Ptriaz-C1-I as a function of temperature. The applied *C*_PIL_s are (c) 5, (d) 10, and (e) 20 mg/mL. Green region: *T* < *T*_c_; Yellow region: *T* > *T*_c_.

Our previous study found that the cloud point temperature (*T*_c_) of Ptriaz-C1-I in methanol was concentration-dependent.^28^ The *T*_c_s of the PIL concentration (*C*_PIL_) at 5, 10, and 20 mg/mL were 27, 45, and 53 °C, respectively. Therefore, we investigated the effects of *C*_PIL_ and the reaction temperature on the catalytic formation of CC3R. First, the reactions were studied at 20, 35, 45, and 60 °C for 1 h at *C*_PIL_s of 0 (without PIL), 5, 10, and 20 mg/mL. The corresponding yields of the CC3R were determined and are summarized in Figure 3b. The yield increased expectedly with increasing reaction temperature, independent of the presence of Ptriaz-C1-I. We observed that at 20 °C the addition of Ptriaz-C1-I did not induce much effect on the reaction. The reason apparently lies in the solution status of catalyst Ptriaz-C1-I, which is below its *T*_c_ and thus is poorly soluble in methanol at 20 °C (see photographs in Figure 3a). Thus, the PIL chains are in a collapsed state at 20 °C and restrict the contact of catalytically active groups (i.e., 1,2,4-triazoliums) with the substrate molecules, so their catalytic effect is largely screened out. At higher temperatures above its *T*_c_, the reaction rates were all significantly higher in the presence of Ptriaz-C1-I than those without Ptriaz–C1-I.

To note, the trend of the difference in the CC3R yield in the presence and absence of Ptriaz-C1-I at varied reaction temperatures is not straightforwardly visible in Figure 3b. For the sake of clarity, the difference between the yields of the reaction with and without the addition of Ptriaz-C1-I at a defined temperature was defined as ΔYield. The ΔYield of CC3R as a function of temperature at *C*_PIL_s of 5, 10, and 20 mg/mL was plotted in Figure 3c–e, respectively. The green region represents the temperature below the *T*_c_ of Ptriaz-C1-I at this PIL concentration, and the yellow region for above its *T*_c_. The polymer chain collapses into a heterogeneous catalyst in the green region and dissolves in solution in the yellow region as a homogeneous catalyst. It was interestingly found that at the same *C*_PIL_, a sharp jump-up in ΔYield occurs at *T* > *T*_c_. For example, the *T*_c_ at *C*_PIL_ ∼ 5 mg/mL was 22 °C, and its ΔYield jumped abruptly from 0.5% (at 20 °C) to 8.9% (at 35 °C). Similarly, the ΔYield of Ptriaz-C1-I over its *T*_c_ jumped sharply from 4.9% (at 35 °C) to 11.1% (at 45 °C) at *C*_PIL_ ∼ 10 mg/mL, and from 7.3% (at 45 °C) to 21% (at 60 °C) at *C*_PIL_ ∼ 20 mg/mL. Obviously, the rate of catalytic CC3R formation is sensitive to the chain conformation of Ptriaz-C1-I in solution in a molecularly dissolved state or in a collapsed state. The catalytic activity is higher when Ptriaz-C1-I is homogeneous (>*T*_UCST_) to expose all the catalytic sites toward substrate molecules, and vice versa, i.e., being heterogeneous (<*T*_UCST_) with the catalytic sites being buried inside the aggregated PIL chains. Note that all of these tests resulted in CC3R with the corresponding structures characterized in Figures S2–S4.

The inverse statistics of the time (*t*^–1^) required to reach a 90% yield of CC3R under different reaction conditions were plotted against *C*_PIL_ in Figure 4a. Three temperatures, 35, 45, and 60 °C, all above their corresponding *T*_c_, were chosen as the reaction conditions to produce CC3R-P5-35, CC3R-P10-45, and CC3R-P20-60, respectively. Results at the same reaction temperature without Ptriaz-C1-I were used as a control. It can be found that, at the same reaction temperature, the reaction rates with Ptriaz-C1-I to reach 90% yield of CC3R are all faster than those without Ptriaz-C1-I due to the catalytic function of Ptriaz-C1-I in this reaction. To quantify the effect, the ratio of these two (with and without Ptriaz-C1-I) was calculated and plotted against the reaction temperature in Figure 4b. The reaction rate increased 1.5-, 2.7-, and 7.5-fold at *C*_PIL_s ∼ 5, 10, and 20 mg/mL at 35, 45, and 60 °C, respectively. This increasing trend is indicative that higher reaction temperatures and Ptriaz-C1-I concentrations promote formation of CC3R.

**Figure 4:**
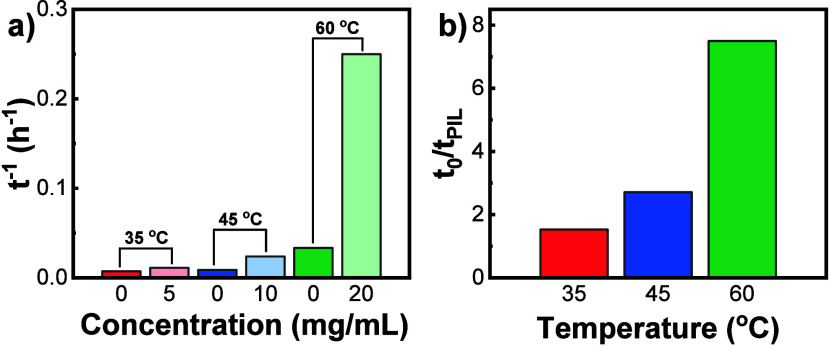
(a) Plot of the inverse statistics of the time (*t*^–1^) under different reaction temperatures and *C*_PIL_ conditions. (b) Plot of ratios of the reaction rate (*t*_0_/*t*_PIL_) in the presence and absence of Ptriaz-C1-I at different reaction temperatures.

The aforementioned data indicate that thermoresponsive Ptriaz-C1-I can indeed modulate the catalytic growth of CC3R through the reaction temperature at defined *C*_PIL_. The Ptriaz-C1-I/methanol system at *C*_PIL_ ∼ 20 mg/mL was chosen as a model to approach the temperature tunability of its catalytic performance. First, we carried out the reaction at 60 °C (>*T*_c_) for 1 h, after which it was cooled down to 0.5 °C (<*T*_c_) for 1 h. This process was repeated thrice. By switching between these high and low temperatures, the yield was measured and plotted against time in Figure 5. There appeared a significant increase in the yield at 60 °C for 1 h, with little-to-no obvious change in the yield at 0.5 °C for 1 h. The same phenomenon was found in the second and third temperature cycles. At 60 °C, Ptriaz-C1-I is soluble in methanol in a homogeneous state with its full power in operation to catalyze the growth of CC3R. At 0.5 °C, the Ptriaz-C1-I chain collapsed, and most of, if not all, catalytically active sites, i.e., 1,2,4-triazolium sites were encapsulated inside the aggregates of polymer chains thus in poor contact with the substrate (Figure 1). As a control, we did an experiment at *C*_PIL_ ∼ 0 mg/mL (without the PIL catalyst, Figure S5). The reaction yield was found to have a similar trend with temperature switching, but the overall reaction rate without Ptriaz-C1-I was much lower. Thus, the temperature-sensitive conformation of Ptriaz-C1-I in methanol can sensitively modulate the catalytic reaction by variation of the reaction temperature.

**Figure 5:**
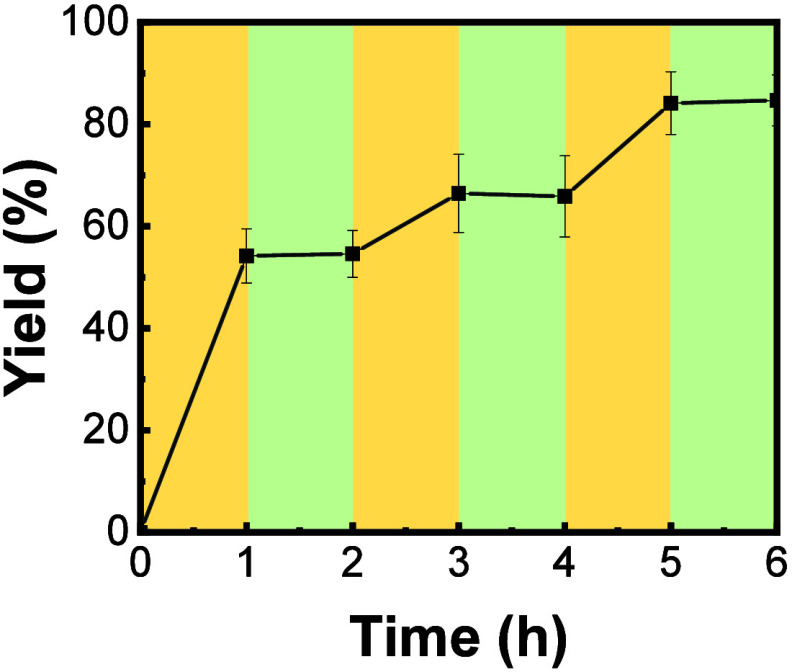
Plot of the yield of CC3R at *C*_PIL_ ∼ 20 mg/mL as a function of reaction time by switching the reaction temperature repeatedly between 60 and 0.5 °C. The yellow and green regions indicate the temperature zone at 60 and 0.5 °C, respectively.

The recyclability of a catalyst is crucial for enhancing the “green nature” of a chemical synthesis. At the end of a model reaction tested at *C*_PIL_ ∼ 20 mg/mL, the resulting CC3R crystals were first collected by filtration at the reaction temperature to leave out a clear filtrate. Then the filtrate temperature was lowered to 20 °C below the *T*_c_ of Ptriaz-C1-I to allow for aggregation and precipitation of the PIL chains. The PIL precipitate was then recovered as heterogeneous precipitates by centrifugation or filtration and could be used in the next reaction. After 6 repeated cycles, Ptriaz-C1-I retained its catalytic performance, as evidenced by a consistent yield after 4 h of reaction (Figure 6), confirming good recyclability and reusability of this thermoresponsive PIL as catalyst.

**Figure 6:**
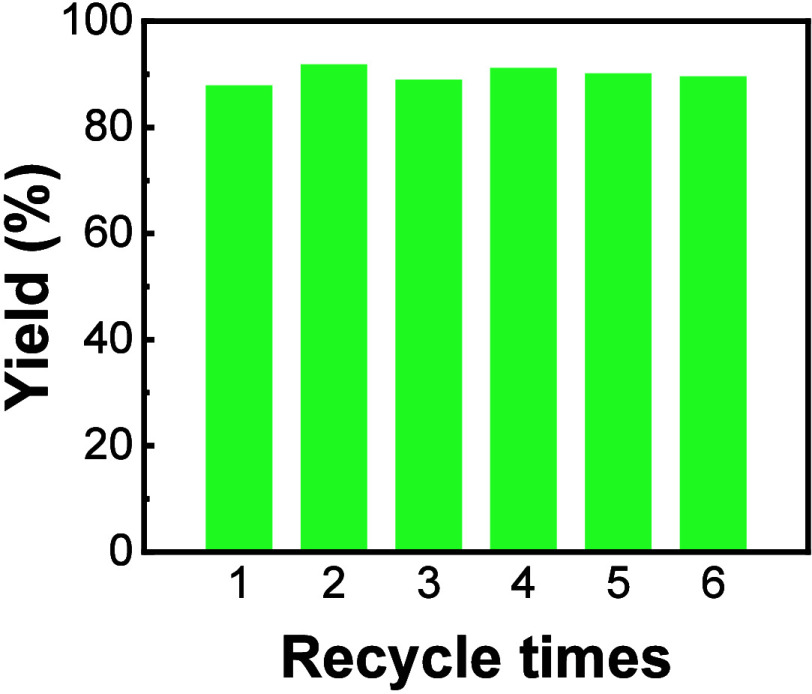
Reusability test of Ptriaz-C1-I in the preparation of CC3R. Each reaction was carried out at *C*_PIL_ ∼ 20 mg/mL at 60 °C for 4 h.

In summary, temperature-modulatable catalysis Ptriaz-C1-I PIL in methanol was investigated for the synthesis of porous organic cage CC3R. This UCST-type thermoresponsive polymer catalyst exhibited distinct catalytic behavior depending on the reaction temperature and its concentration-dependent cloud point temperature. At temperatures above its *T*_c_, the polymer chains were fully dissolved, ensuring excellent substrate contact with the catalytically active sites and enabling efficient homogeneous catalysis. Conversely, at temperatures below *T*_c_, the polymer chains collapsed and aggregated, encapsulating the active sites and retarding the reaction. This phase transition also shifts the catalyst from a homogeneous state to a heterogeneous state, allowing for straightforward recovery and reuse. This study on thermoresponsive organocatalysts offers a model system to study the fine control of the catalytic process and is inspiring for the design of “smart” devices in chemical reaction engineering.

## Supporting Information Available

Experimental section and supplementary characterization data (N_2_ adsorption–desorption curves, powder XRD, ^1^H NMR, FT-IR) (PDF)
