# Supplementary material for: Reversible Switching and Recycling of Thermoresponsive 1,2,4-Triazolium-Based Poly(ionic liquid) Catalysts for Porous Organic Cage Synthesis in Organic Media
Source: ACS Macro Lett. 2025 Mar 24;14(4):458–63. doi: 10.1021/acsmacrolett.5c00072 (PMC12004921; doi:10.1021/acsmacrolett.5c00072)
Supplement: Supplementary file 1 — mz5c00072_si_001.pdf [file mz5c00072_si_001.pdf]

Supporting information

Reversible Switching and Recycling of Thermoresponsive 1,2,4-Triazolium-based Poly(ionic Liquid) Catalysts for Porous Organic Cage Synthesis in Organic Media

Jiefeng Zhu,[1] Feng Chen,[1,2] Jie Zhang,[1] Ruijie Hou,[1] Jian-ke Sun, [3] Xianjing Zhou,[1*] Jiayin Yuan,[4*] Xinping Wang[1*]

[1] School of Chemistry and Chemical Engineering, Key Laboratory of Surface & Interface Science of Polymer Materials of Zhejiang Province, Zhejiang Sci-Tech University, Hangzhou 310018, China

[2] State Key Laboratory for Modification of Chemical Fibers and Polymer Materials, College of Materials Science and Engineering, Donghua University, Shanghai 201620, China

[3] School of Chemistry and Chemical Engineering, Beijing Institute of Technology, Beijing, 102488, China

[4] Department of Materials and Environmental Chemistry, Stockholm University, Stockholm 10691, Sweden

[*] Corresponding author.
Xianjing Zhou (E-mail: xjzhou@zstu.edu.cn), Jiayin Yuan (E-mail: jiayin.yuan@mmk.su.se) & Xinping Wang (E-mail: wxinping@zstu.edu.cn)

1.Experimental section

1.1. Chemicals.

1,3,5-Triformylbenzene (96%), (*R,R*)-1,2-diaminocyclohexane (98%), methanol (AR), ethanol (AR), and dichloromethane (AR) were purchased from commercial sources and used without further purification. The preparation process and structural characterization of Ptriaz-C1-I are detailed in our previous paper.[1]

1.2. Synthesis of porous organic cage CC3R catalyzed by PIL.

1,3,5-Triformylbenzene (30 mg, 0.19 mmol), (*R,R*)-1,2-diaminocyclohexane (32 mg, 0.28 mmol) and a defined amount of Ptriaz-C1-I were dissolved in 1, 0.5 and 1.5 mL of methanol, respectively. The above three solutions were quickly mixed and reacted at a defined temperature. After the reaction, the solid product grew on the vessel wall and was separated by scratch and filtration. The crystals were washed with an ethanol/dichloromethane mixture (95:5, v:v) and dried under vacuum at 70 °C overnight to give the product.

1.3. Synthesis of porous organic cage CC3R without PIL.

The synthesis and purification procedure of CC3R was consistent with the method mentioned above, except that Ptriaz-C1-I was not added to the reaction system.

1.4. Characterization.

Powder X-ray diffraction (XRD) data were collected on an A8 Avance (Bruker) instrument. The samples were measured at a speed of 5 °/min with an angular range of 5° to 90°. Proton nuclear magnetic resonance (^{1}H NMR) spectra were conducted on an MRI (400 MHz, Bruker) spectrometer. Fourier transform infrared (FT-IR) spectra were recorded on a Nicolet iS10 spectrometer (Thermo Fisher Scientific). Reflection mode optical microscope (OM, LV100-POL type, NIKON Japan) with a microscope camera (DS-Fi3 type, NIKON, Japan) and scanning electron microscopy (SEM, RegulusSU8100, Japan) were used to characterize the morphology of CC3R. Specific surface area was conducted by Autosorb-iQ fully automated specific surface and pore size distribution analyzer. The cloud point temperature (T_c) of Ptriaz-C1-I/methanol solutions at different concentrations were measured by a temperature-variable UV−vis

spectrophotometer (Cary300 UV, Agilent, USA). The phase diagram was plotted with T_c as y-axis and volume fraction of Ptriaz-C1-I as x-axis. For ease of volume fraction calculation, the density of Ptriaz-C1-I was estimated to be 1 g/mL. Limited by the solubility of Ptriaz-C1-I in methanol and the boiling point of methanol (*i.e.*, 64.7 ^{o}C), it was not possible to present a complete phase diagram.

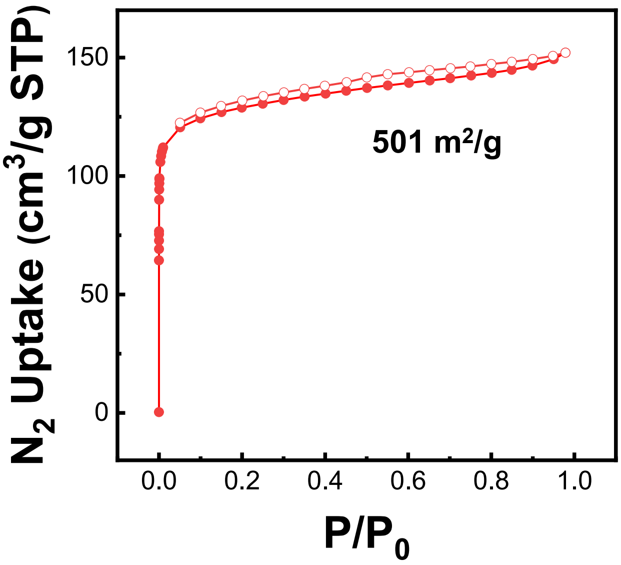

Figure S1. N_2 adsorption-desorption isotherms of CC3R-P20-60. The sample was degassed at 80 ^{o}C for 12 h under vacuum before analysis at 77 K (N_2).

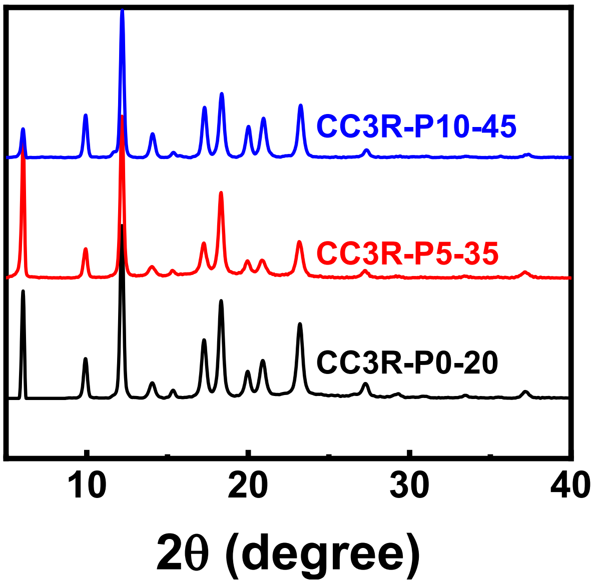

Figure S2. Powder X-ray diffraction patterns of CC3R-P0-20, CC3R-P5-35, and CC3R-P10-45.

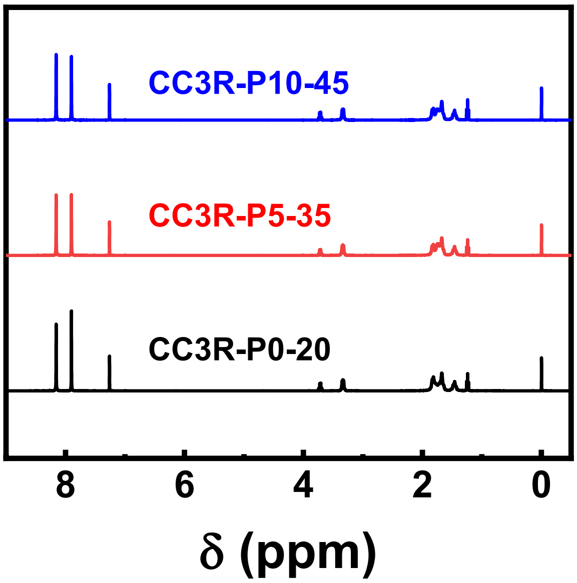

Figure S3. ^{1}H NMR spectra of CC3R-P0-20, CC3R-P5-35, and CC3R-P10-45.

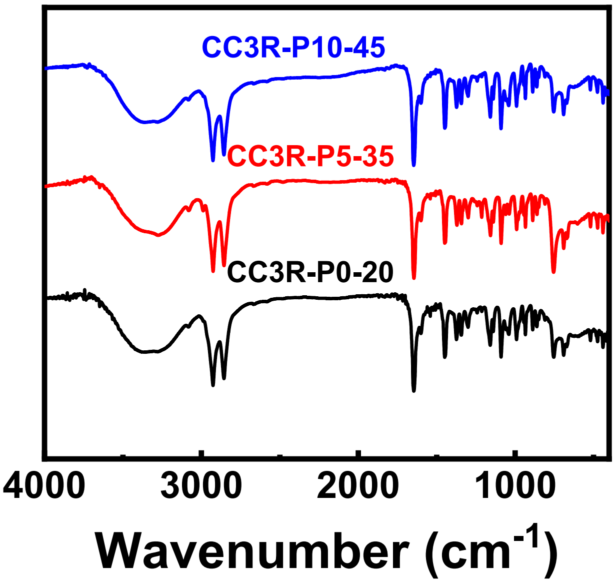

Figure S4. FT-IR spectra of CC3R-P0-20, CC3R-P5-35, and CC3R-P10-45.

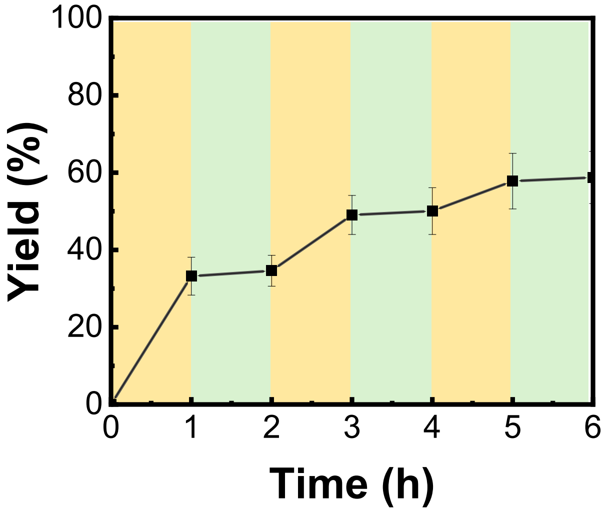

Figure S5. The plot of the yield of CC3R at $C_{\mathrm{PIL}} \sim 0$ mg/mL (without PIL) as a function of reaction time by switching the reaction temperature repeatedly between 60 ^{o}C and 0.5 ^{o}C. The yellow and green regions indicate the temperature zone at 60 ^{o}C and 0.5 ^{o}C, respectively.

References

(S1) Chen, F.; Zhu, J.; Hou, R.; Zhou, X.; Yuan, J.; Wang, X. Thermal responsiveness of 1,2,4-triazolium-based poly (ionic liquid) s and their applications in dye extraction and smart switch. *ACS Appl. Polym. Mater.* **2024**, *6*, 13202-13209.
